# Integrating Patient Safety Education in the Undergraduate Nursing Curriculum: A Discussion Paper

**DOI:** 10.2174/1874434601812010125

**Published:** 2018-06-29

**Authors:** Mansour J Mansour, Shadi F Al Shadafan, Firas T Abu-Sneineh, Mohammed M AlAmer

**Affiliations:** 1 Fundamentals of Nursing Department, College of Nursing, Imam Abdulrahman Bin Faisal University, Dammam, Saudi Arabia; 2 Community Health Nursing Department, College of Nursing, Imam Abdulrahman Bin Faisal University, Dammam, Saudi Arabia

**Keywords:** Patient Safety, Nursing Education, Undergraduate Curriculum, WHO, Modern model

## Abstract

**Background:**

This paper explores the opportunities and challenges for integrating patient safety education in undergraduate nursing curriculum.

**Methods:**

Four dimensions of undergraduate nursing education are examined: National accreditation of nursing programs, building a competency-based nursing education, a model of nursing education and building faculty capacity in patient safety education and research.

**Results:**

Incorporating patient safety in a nursing curriculum can be “institutionalized” by making it a pre-requisite for granting program accreditation. At the operational level, transforming undergraduate nursing education to incorporate inquiry-based learning and moving toward competency-based patient safety education are two key requirements for engaging the students with patient safety science. Building faculty capacity who are experts in both patient safety teaching and research remains a key challenge that needs to be addressed to enable a shift in the patient safety “mindset” for future nursing workforce.

**Conclusion:**

Efforts to introduce patient safety in nursing education are both necessary and timely, and should accommodate students’ unique needs and cultural context.

## 1 INTRODUCTION

Patient Safety is a growing global phenomenon. *Do no Harm* is a basic ethical principle that underpins health care delivery. Patient safety education for the healthcare workforce has emerged as an important tool in safeguarding patient welfare [1]. Evidence from the literature suggests that early engagement of nursing students with patient safety principles has a significant impact on developing and shaping their long-term patient safety knowledge, skills and behaviors [2]. Although the basic pillars which underpin modern patient safety thinking are now recognized as integral part of health professional education, there is still ambiguity on how patient safety can be best incorporated into both clinical and theoretical health care education, including in nursing [3].Globally, efforts to address rising patient safety concerns, medical litigations and consumer pressure have been pursued by health care organizations, both governmental and non-governmental, to address gaps in patient safety and care delivery. Several initiatives have been implemented, including establishing patient safety standards and addressing patient safety goals as part of a hospital accreditation process as well as investing in staff training [4]. Undergraduate nursing education represents an important venue for providing future generations of nurses with the patient safety skills fit for the 21^st^ century. The aim of this paper is to discuss the current opportunities for integrating patient safety education in the undergraduate nursing education and potential challenges that are likely to hinder successful integration.

## 2 SETTING THE CONTEXT

Since the movement of patient safety started toward the end of the 20^th^ century with the publication of the Institute of Medicine (IOM) landmark report *To Err is A Human* [5], there has been a conspicuous drive to bring into light strategies and solutions to manage patient safety risk. Although patient safety has been made a priority in many counties, many patient safety practices, such as team work, safe cross-discipline communication skills and reporting of unsafe practice, are yet to be fully integrated and practiced effectively among health care professionals [6].

Patient safety has been at the forefront of the international efforts to raise awareness and safety of healthcare delivery. Many hospitals have been achieving Magnet^®^ hospital status or accreditation by The American Nurses Credentialing Center (ANCC). Obtaining Magnet hospital status is a prestigious achievement for any health care organization, and it implies the fulfillment of certain criteria within the healthcare organization that are paramount for establishing patient safety culture. For example, one of the criteria is establishing a “just culture” where professional accountability and error reporting are supported by the upper echelon of the health care organization [7]. Solid evidence must be submitted with the Magnet application to back up the fulfillment of this criterion. So when employing registered nurses who have already been taught on relevant and contemporary patient safety science in their undergraduate nursing education, achieving Magnet status will be less challenging, but it will also be more beneficial for raising the profile of patient safety among the nursing workforce and the wider organizational context. Successful achievement of the Magnet designation demonstrates a sizeable degree of achieving patient safety and quality standards that are, in many ways, comparable to those in the US. It was suggested that implementing broader changes in the team and organization should be coupled with recognizing the contributions of education and training toward developing a patient safety-oriented workforce, and by eliciting changes in healthcare providers’ knowledge, skills and attitudes [8]. The nursing workforce represents, by far, the largest segment of professionals working in the healthcare industry [9]. And even though there is a wealth of evidence in the literature on strategies to improve patient safety and quality of care, the vast majority of healthcare professionals are not adequately educated in patient safety. This results not only in subsequent missed opportunities for knowledge dissemination but also in inadequate translation of existing knowledge into tangible practice [10]. Therefore, embedding patient safety in nursing education is bound to occupy a considerable volume of discussion.

## 3 NATIONAL ACCREDITATION OF UNDERGRADUATE NURSING CURRICULUM

Academic Accreditation of a nursing program is a significant milestone in any academic institution that provides nursing education. To grant accreditation, the nursing education provider must meet certain criteria to ensure the robustness of the quality content, delivery and nursing education but also that proper system-checks are in place.

There are good exemplars of international educational systems where patient safety integration was carried out with reasonable success despite logistical challenges. This is notably happening in the US, UK and Australia. However, embedding patient safety in undergraduate nursing education still faces many challenges in these countries and elsewhere. A national report which examined the status of patient safety education in UK undergraduate healthcare curriculum found that both students and academic staff perceived the lack of mandatory coverage of patient safety curriculum to limit formal exposure to education and training for patient safety [8]. There are many opportunities that, if exploited appropriately, can provide a significant leap in improving patient safety skills, knowledge, and attitudes of future nursing workforce. The national academic accreditation bodies which are tasked with safeguard academic standards in educational institutions take the lead in these efforts. A core mission of these academic entities is to ensure that the quality of education and administration in higher education institutions keeps pace with the high international standards. Those academic regulators can play a vital role in facilitating structured integration of patient safety education in undergraduate nursing curriculum by making it mandatory to include important elements of patient safety education, such as Human Factors, into national nursing curriculum as well as the related evidence for effective teaching strategies. This approach has already been recommended by the World Health Organization (WHO) ‘Patient Safety Program’ [1], and was utilized successfully in the US [11], UK [12] and Australia [13]. In other countries, there may have been an appreciation of the critical role of education and training in patient safety, but often, such appreciation has not been translated into tangible steps to address the challenges that face full integration of patient safety education in healthcare curriculum [3]. It was suggested that any training of patient safety that is tailored to the healthcare profession, including Human Factors and non-technical skills, needs to start early in the undergraduate nursing program. It may be too late to deliver this training toward the end of the program, or after the completion of the undergraduate education, when professional attitudes are almost fully-developed [14], [16]. We would agree that adopting patient safety skills, knowledge and attitude in the undergraduate nursing education as part of accreditation process would help to “institutionalize” teaching patient safety at a national level, particularly for those colleges of nursing which are seeking the academic accreditation for the first time, where the academic staff may have a fresh start with a new curriculum, thus allowing them to acquire more easily new patient safety knowledge, skills and attitudes, without the need to amend old, deeply-rooted ones. Developing and accrediting Bachelor nursing program dictate that the program must be “…delivering safe, evidence-based care to patients, families, and groups, in a variety of care settings [10].” Lessons can be learnt from other countries where patient safety education has already been introduced as a pre-requisite for granting academic nursing accreditation; however, how this can be operationalized into actual teaching topics and delivery methods is less clear, taking into consideration the unique cultural and educational contexts for each academic setting.

## 4 TOWARD COMPETENCY-BASED NURSING EDUCATION FOR PATIENT SAFETY

While there is a consensus on the importance of integrating patient safety education in undergraduate nursing curriculum, it is equally essential to agree on the operational definitions of these specific subsets of the patient safety in educational pedagogy. For example, what patient safety topics/skills need to be adopted and taught in any future curriculum mapping? What are the best teaching pedagogies that need to be selected and implemented? All these are legitimate questions that must be carefully considered when designing and implementing any future patient safety-friendly nursing curriculum.

Internationally, there are two patient safety frameworks which have been published in recent years and are being used as a “road-map” for developing and incorporating patient safety teaching in health care curricula, including nursing. Both frameworks aim at challenging the assumption that only incompetent nurses make mistakes and engage in improving system safety later in their professional career. The first one is the WHO’ Multi-Professional Patient Safety Curriculum Guide [1], which was published in 2011, and the second is the Quality and Safety Nurse Education Framework (QSEN) [11], which was developed in the US specifically for undergraduate nursing curriculum. The WHO’ Multi-Professional Patient Safety Curriculum Guide contains eleven patient safety themes (Table **1**).

The WHO launched the Multi-Professional Patient Safety Curriculum Guide in the Middle East in a workshop which was held at Imam Abdulrahman Bin Faisal University - College of Nursing in April 2013, (Formarly known as University of Dammam) affirming the drive for incorporating patient safety education in nursing curriculum in the Middle Eastern Region and beyond. The Guide affirms the need to include theses teaching topics in the nursing curriculum but with less clear considerations on the best way to teach them. Subsequent international evaluation of this Guide by both the faculty and students highlighted its effectiveness in teaching patient safety. In addition, its content was, overall, culturally appropriate for their country [14]. By contrast, the traditional teaching methods recommended by this Guide WHO were not always effective in conveying a concrete change in the nursing students’ knowledge, skills and attitudes in relation to the patient safety topics taught [15].Nonetheless, the suggested topics are undoubtedly important in raising the awareness of modern thinking of patient safety among nursing students and faculty members alike, rendering them better prepared to manage the challenging patient safety issue in practice.

The QSEN aims at transforming nursing education through the integration of six core-based Knowldge, Skills and Attitudes (KSA) attributes for each competency. These competencies are: patient-centered care, teamwork and collaboration, evidence-based practice, quality improvement, safety and informatics [11].The QSEN provides a detailed mapping for each core competency with sound explanation to the relevant KSA of each competency. Although the empirical testing of these core competencies in the nursing curriculum was not problem-free [18], there is a prevailing argument in the literature for integrating these competencies as a new way for structured integration of patient safety science in undergraduate nursing education [17, 20]. The need to ensure that the undergraduate nursing education has explicit and relevant elements of patient safety is timely, and while the academic accreditation regulators may dictate the nursing education provider to integrate certain elements of patient safety in their curricula, we suggest that it is up to the applying higher education institution to translate such requirements into tangible educational steps in their nursing curriculum. To our knowledge, there are no further frameworks which guide the higher education institution to translate such requirements into a more operational context. It is imperative that any attempt to adopt international frameworks in patient safety, whether it is the WHO Patient Safety Multi-Curriculum Guide or the US QSEN path, will need to be tailored to the unique cultural and educational contexts for each academic setting.

Collaboration among all the undergraduate nursing education providers is essential to standardize patient safety education or at least to draft a wide consensus on what elements must be addressed in any future consideration of patient safety-friendly nursing curriculum. Both undergraduate and graduate nurses need skill sets identified and agreed upon by all stakeholders, and there should be a mechanism to maintain a close feedback loop on this issue. A commitment by influential stake holders, regulators and professional bodies is an essential step to develop and sustain an effective patient safety education for future nursing workforce.

## 5 MODERN MODEL FOR NURSING EDUCATION

A key pre-requisite for embedding patient safety education in higher education institutions is the transformation of the teaching methods to incorporate better students’ engagement. There is an international drive, and perhaps a need, that nursing education must be aligned with Inquiry-Based Learning (IBL) approach, which is commonly used in many higher education institutions and is now being accepted as an ideal teaching strategy in nursing education [21]. IBL is the process of learning in which learners have a greater control in selecting the choice of aim, scope or topic of their learning [22]. To learn more effectively, the IBL approach also dictates the students to master a set of interpersonal and learning skills, including team working, information gathering, communication and most importantly, high order cognitive skills for professional development – which are highly valued by most employers [23].This dictates a shift in the “educational mindset”, where the faculty members are no longer the only source of information that the students may pursue, and the faculty must accept this fact, particularly when discussing problems that are formulated within complex, inter-professional dimensions [22].

In the context of patient safety education, these transferable skills of inquiry are paramount for the 21^st^ century employability economy, and are likely to facilitate deep learning approach, as opposed to surface or strategic learning ones [24]. The traditional form of university teaching style that is largely dependent on the use of conventional, face-to face lecture, discourages active student involvement, and pushes for increased student passivity [25].On the contrary, teaching which involves students as active and independent learners is more likely to encourage a deep learning approach, where students engage in the task in a more meaningful manner [26]. Integrating a modern way of teaching based on more student engagement, facilitated group discussion and flipped classroom demonstrated a better student engagement, and subsequently, a better information-retention rate of knowledge and application [27]. While this can be true across a broad spectrum of disciplines and health programs, it is of particular benefit for patient safety education. Patient safety science is said to be multi-facets, with combinations of knowledge, skills and attitudes [11]. To achieve the best teaching and learning environment, diverse teaching strategies will have to be utilized both in the classroom and clinical placement settings. This helps to move away from the traditional spoon-feeding teaching strategy that promotes surface and strategic learning approach to a more student-oriented manner rooted in deep learning approach. A classical example for using IBL is with safe communication and decision making in critical situations. Students are often presented with challenging and complex patient scenarios, where they should gather information, analyze it and apply the best outcome based on information solicited from diverse information outlets.

Adopting IBL in nursing education, and along with patient safety one, is not challenge-free. Academics consider this approach to be laborious and resource-intensive [22], but more fundamentally, they may struggle to come to terms with students who are unable to handle hypothetical problems without being “given foundations” [28].For those universities which still use the spoon-feeding teaching style in nursing education, both students and faculty are likely to struggle to appreciate the ethos behind using IBL, but may also fail to value the opportunity of sharing experiential learning and common goals, and the importance of multi-disciplinary teamwork skills on the longer term, particularly if the concept of Inquiry-Based nursing was introduced to them without adequate foundational knowledge. Worldwide, the use of this innovative teaching method in higher education has become more visible in recent years, but the pace of its integration in nursing curricula is proven to be more challenging, including the lack of support and training for both faculty and student alike [29]. The feelings of uncertainty of the effectiveness of this approach as learning framework, as well as impact of culturally determined focus on tradition, where students referred to their society’s respect for the ‘old ways’ and wariness regarding innovations [30]. Many universities from less able countries have already started to overcome these challenges by forging academic partnerships with other universities in the US, UK and Australia, where the IBL approach is well-established in their nursing curricula. Such partnerships are likely to foster knowledge transfer to the destination institutions, but will also nurture the development of home-growing faculty expertise in the development, delivery and evaluation of IBL in nursing curriculum.

There is a huge potential for the use of mobile and e-learning technology in patient safety education in nursing curriculum. Prospective students who are entering higher education are increasingly becoming digital natives [31], and this is likely to have conspicuous pedagogical implications on the undergraduate nursing curriculum. On one side, the students are becoming heavy users of mobile devices and other information technology platforms, and this provides excellent opportunities for delivering nursing education that is engaging, fit for purpose and able to transform the way patient safety is taught. Mobile technologies are being introduced into nursing education to address the challenges that students face in both theory and clinical settings [32]. Studies among university students reported that 82% to 95% of the students possess smartphones [33], [34]. Such a prolific use of mobile devices represents an excellent opportunity to engage students in using these devices. Many instant messaging educational app have become very popular for classroom teaching such as Nearpod^®^, Socrative^®^ and Padlet ^®^. And if used effectively, they will have a huge potential to enhance the learning process of students, including nursing ones.

## 6 CAPACITY BUILDING FOR PATIENT SAFETY EDUCATION

One of the challenges that face the successful integration of patient safety in nursing curriculum is the lack of competent educators who are familiar with teaching modern patient safety concepts based on system-wide knowledge and learning. It was reported that patient safety teaching in undergraduate nursing curriculum was not explicitly linked to patient safety agenda, or at the best was not explicit in the formal curriculum [35], [36]. it is noteworthy that understanding the patient needs within the students’ unique educational and cultural contexts is pivotal for delivering quality patient safety education. While there are common principles of patient safety science across all health care disciplines, the core aspiration of training interventions is to “elicit change” and to address culture sensitivity adequately. For example, male and female nursing students are separated in different campuses in most Saudi educational institutions. This may represent a challenging situation, particularly when there is a shortage of male and female faculty members who are well-trained to deliver teaching on specialized patient safety topics (*i.e.* Human Factors). Train–the trainer approach [37] in Human Factors and patient safety skills may be one way for overcoming this challenge and at the same time saving extra cost for additional faculty recruitment.

Research into patient safety education, in nursing and other health care curricula, is an important area that must be given a considerable support if substantial progress in error-reduction strategies is to be taught, developed and sustained. Further research is needed to better understand how the design, teaching and delivery of patient safety education can be tailored to each educational system and discourse. Obviously, funding for such research is paramount for developing research capacity, expertise and infrastructure for patient safety education.

## CONCLUSION AND THE WAY FORWARD

This discussion paper aimed at outlining few opportunities and challenges that face patient safety education in undergraduate nursing curriculum, and the discussion is by no means inclusive of all issues. Rather, the discussion focused on few aspects which the authors felt they are of important relevance to integrating patient safety education in undergraduate nursing curriculum.

Patient safety education of health care professionals has neither kept pace with advances in patient safety nor with workforce requirements. The introduction of patient safety education in undergraduate nursing curriculum is therefore necessary and timely. Evidence from the literature emphasized the significant impact of early exposure of undergraduate students to basic concepts in patient safety and error-reduction strategies. This is essential to shift the mindset of safe care delivery for future nursing workforce, where they are well-prepared for collaboration and inter-professional teamwork, and who are able to adapt to local and cultural needs. There is a huge potential for transforming patient safety education in undergraduate nursing education, and while there are many challenges ahead, many opportunities do also exist. Notably, the accreditation of undergraduate nursing program can play a vital role in “institutionalizing” system-based patient safety education in nursing curriculum at national level by making it a mandatory requirement for the colleges of nursing to integrate elements of patient safety in their curriculum when seeking national accreditation. This is likely to signify the commitment to patient safety for the faculty, students and the wider community. While there are leading examples on adopting such a patient-safety friendly curriculum in the US, UK and Australia, many other countries are required to follwo suit. Operationally, two patient safety education frameworks that can be consulted when designing patient safety-friendly nursing curriculum are: the WHO Multi-Professional patient safety curriculum guide and the QSEN framework. More empirical research is needed to inform the policy-makers on the development and integration of operational definition of patient safety education in nursing and healthcare disciplines. Leotsakos et al [3] from the WHO Patient Safety Program argued that “Patient safety is not another subject to add to an already over-packed curriculum, but serious thought is required as to how health professional teachers can integrate patient safety competencies into their clinical teaching and learning.” For successful patient safety cultural change, health care professionals should strive to accommodate both workforce unique needs and limitations.

## Figures and Tables

**Table 1:** World Health Organization Patient Safety Curriculum Guide: Multi- profession Edition 2011.

1. What is patient safety? 2. Why applying human factors is important for patient safety. 3. Understanding systems and the effect of complexity on patient care. 4. Being an effective team player 5. Learning from errors to prevent harm 6. Understanding and managing clinical risk 7. Using quality improvement methods to improve care 8. Engaging with patients and carers 9. Infection prevention and control 10. Patient safety and invasive procedures 11. Improving medication safety
